# Two New Pimelic Diphenylamide HDAC Inhibitors Induce Sustained Frataxin Upregulation in Cells from Friedreich's Ataxia Patients and in a Mouse Model

**DOI:** 10.1371/journal.pone.0008825

**Published:** 2010-01-21

**Authors:** Myriam Rai, Elisabetta Soragni, C. James Chou, Glenn Barnes, Steve Jones, James R. Rusche, Joel M. Gottesfeld, Massimo Pandolfo

**Affiliations:** 1 Laboratoire de Neurologie Expérimentale, Hôpital Erasme, Brussels, Belgium; 2 Department of Molecular Biology, The Scripps Research Institute, La Jolla, California, United States of America; 3 Repligen Corporation, Waltham, Massachusetts, United States of America; Hospital Vall d'Hebron, Spain

## Abstract

**Background:**

Friedreich's ataxia (FRDA), the most common recessive ataxia in Caucasians, is due to severely reduced levels of frataxin, a highly conserved protein, that result from a large GAA triplet repeat expansion within the first intron of the frataxin gene (*FXN*). Typical marks of heterochromatin are found near the expanded GAA repeat in FRDA patient cells and mouse models. Histone deacetylase inhibitors (HDACIs) with a pimelic diphenylamide structure and HDAC3 specificity can decondense the chromatin structure at the *FXN* gene and restore frataxin levels in cells from FRDA patients and in a GAA repeat based FRDA mouse model, KIKI, providing an appealing approach for FRDA therapeutics.

**Methodology/Principal Findings:**

In an effort to further improve the pharmacological profile of pimelic diphenylamide HDACIs as potential therapeutics for FRDA, we synthesized additional compounds with this basic structure and screened them for HDAC3 specificity. We characterized two of these compounds, **136** and **109**, in FRDA patients' peripheral blood lymphocytes and in the KIKI mouse model. We tested their ability to upregulate frataxin at a range of concentrations in order to determine a minimal effective dose. We then determined in both systems the duration of effect of these drugs on frataxin mRNA and protein, and on total and local histone acetylation. The effects of these compounds exceeded the time of direct exposure in both systems.

**Conclusions/Significance:**

Our results support the pre-clinical development of a therapeutic approach based on pimelic diphenylamide HDACIs for FRDA and provide information for the design of future human trials of these drugs, suggesting an intermittent administration of the drug.

## Introduction

Friedreich's ataxia (FRDA) is the most common of the early-onset autosomal recessive ataxias in Caucasians. In addition to progressive neurological disability, FRDA causes a hypertrophic cardiomyopathy and an increased risk of diabetes mellitus. Skeletal abnormalities such as kyphoscoliosis and pes cavus are common. The first symptoms usually are noticed around the time of puberty [1], [2]. As is the case for almost all neurodegenerative diseases, no proven treatment that can stop the progression of FRDA is now known. FRDA is caused by severely reduced levels of frataxin [3], a highly conserved mitochondrial protein, that result from a large GAA triplet repeat expansion within the first intron of the frataxin gene (*FXN*). *In vitro* and *in vivo* in bacterial plasmids the expanded repeat can adopt a triple helical structure that directly interferes with transcriptional elongation [4]. However, the discovery that long GAA repeats suppress the expression of a nearby reporter gene in transgenic mice in a manner similar to position effect variegation (PEV) observed in Drosophila pointed to a role of epigenetic mechanisms in the pathogenesis of FRDA [5], [6], [7]. PEV results in the silencing of a gene located near a heterochromatic region because of the spreading of heterochromatin into the gene itself. This phenomenon does not occur in all cells, hence the term “variegation”, but nevertheless leads to an overall downregulation of the involved gene at the tissue and organ level. In agreement with this hypothesis, the typical marks of heterochromatin, such as DNA methylation and histone deacetylation, are found near the expanded GAA repeat both in FRDA patients' cells and in mouse models [6], [7], [8], [9]. Based on these observations, we speculated that histone deacetylase (HDAC) inhibitors (HDACIs) might reverse *FXN* silencing by directly increasing histone acetylation on the *FXN* gene, leading to chromatin decondensation and active transcription. The dynamic interplay between histone acetylation, performed by histone acetyltransferases (HATs) and deacetylation, catalyzed by HDACs, is indeed a central mechanism to regulate gene expression, with increased acetylation associated with an open chromatin conformation and active genes [10]. Eighteen HDACs (more strictly, protein deacetylases) have been identified in the human genome, including the zinc-dependent HDACs (class I, class II, and class IV), and the NAD^+^-dependent protein deacetylase enzymes (class III, or sirtuins) [11]. Accordingly, a diverse class of compounds that inhibit HDACs has been developed. Despite a potential widespread effect of HDAC inhibition on key processes as cellular differentiation and development, many of these compounds are well tolerated in humans and some of them show therapeutic promise in a wide range of diseases, including cancer, metabolic and neurological diseases [12], [13]. Target specificity and kinetic properties of HDACIs probably define the spectrum of genes whose expression they affect, explaining why a catastrophic general deregulation of gene expression is not observed with their use.

We found that a commercially available HDACI (BML-210), and derivatives we have synthesized (pimelic diphenylamides), relieve repression of the *FXN* gene in lymphoid cell lines derived from FRDA patients, in primary lymphocytes from donor FRDA patient blood, and in the brain and heart of a mouse model for FRDA [8], [9]. Unexpectedly, we also found that only members of the pimelic diphenylamide family of HDAC inhibitors increase *FXN* gene expression, and many common and highly active HDAC inhibitors, including the hydroxamates trichostatin A (TSA) and suberoylanilide hydroxamic acid (SAHA), are inactive. Pimelic diphenylamide HDACIs specifically target class I HDACs, with the highest inhibitory effect on HDAC3 [14]. They are further characterized by a slow-on slow-off kinetics, leading to a more persistent histone hyperacetylation than induced by other HDACIs, including very potent molecules like suberoylanilide hydroxamic acid (SAHA).

In an effort to further improve the pharmacological profile of pimelic diphenylamide HDACIs as potential therapeutics for FRDA, we synthesized additional compounds with this basic structure and screened them for HDAC3 specificity. Here we report the characterization of two of these compounds in FRDA patients' peripheral blood lymphocytes and in the KIKI mouse model. Our results support the development of a therapeutic approach based on pimelic diphenylamide HDACIs for FRDA and provide information for the design of future human trials of these drugs [14].

## Results

### Two Novel Pimelic Diphenylamides with Histone Deacetylase Inhibitor Activity

**Figure 1:**
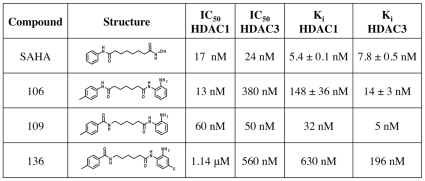
Structure and kinetic properties of histone deacetylase inhibitors. The structures of the new compounds **109** and **136** are shown, along with the structures of the previously described compound **106** [9] and of SAHA. For each compound, the IC_50_ and K_i_ for HDAC1 and HDAC3 are provided.

Two compounds, similar in structure to each other and to compound **106** *N* ^1^-(2-aminophenyl)-*N* ^7^-*p*-tolylheptanediamide), but having a different pattern of HDAC isotype inhibition, were synthesized: **109:** *N-(6-(2-aminophenylamino)-6-oxohexyl)-4-methylbenzamide* and **136** *N-(6-(2-amino-4-fluorophenylamino)-6-oxohexyl)-4-methylbenzamide* (Figure 1). Purified recombinant HDAC enzymes were used with a fluorescent substrate to determine IC_50_ and K_i_ values (see Methods S1). The K_i_ values for HDAC1 and HDAC3, given as a ratio of HDAC1/HDAC3 are: ∼6 fold for **109** and ∼3 fold for **136** Figure 1). Both molecules showed the same slow-on/slow-off inhibition kinetics previously demonstrated for other compounds of the same class (Figures S1 and S2) [15].

### Effects of Compounds 136 and 109 in Unstimulated Peripheral Blood Mononuclear Cells from FRDA Patients

**Figure 2:**
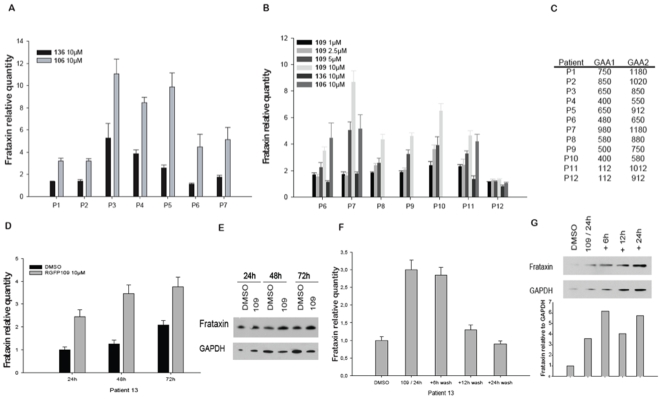
Effect of compounds 136 and 109 on FRDA patients' primary lymphocytes. **A.** *FXN* mRNA levels were measured in RNA from isolated PBMCs from patients (P1–P7) after a 48-hour incubation with either **136** or **106** at 10 µM or DMSO (at 0.1%). Quantitative real-time RT-PCR was used to determine relative *FXN* levels for each patient condition using GAPDH as a control housekeeping gene. DMSO condition was set to value 1 for each patient. Error is represented as the standard deviation of the mean from 3 determinations. *p*<0.001 in all tested patients with compound **106**. Primary lymphocytes were obtained from donor blood from FRDA patients as described [8]. **B.** *FXN* mRNA measurement by quantitative real-time RT-PCR in RNA from isolated PBMCs from patients (P6–P12) after a 48-hour incubation with **109** at 1, 2.5, 5 or 10 µM, or **106** at 10 µM or or **136** at 10 µM or with DMSO (at 0.1%) as control. DMSO condition was set to value 1 for each patient, with GAPDH as reference gene. *p*<0.001 for P6-7 with compound **109** at 5 and 10 µM and with compound **106**; *p*<0.001 in all conditions for P8-P11 except for P11 condition with compound **136**; *p*<0.001 for P12 only with compound **109** at 10 µM only. **C.** Representative table of GAA repeat size in tested patients samples. **D.** PBMCs from patient P13 were treated with either **109** at 10 µM or DMSO for 24, 48 or 72 hours before harvesting. *FXN* mRNA was determined by quantitative real-time RT-PCR, relative to GAPDH. **E.** Protein extracts were prepared from harvested cells from panel D. treated with DMSO or **109**, and subjected to western blotting with frataxin or GAPDH antibodies. **F.** Another extraction of PBMC from patient P13 were treated with either **109** at 10 µM or DMSO for 48 hours and harvested after a 6, 12 or 24 hours wash out period. *FXN* mRNA was determined by real-time RT-PCR and in **G.**, frataxin protein was assessed by western blotting followed by densitometry quantification after the same time points for wash out.

We tested compounds **136** and **109** for their ability to upregulate frataxin mRNA in cultures of unstimulated peripheral blood mononuclear cells (PBMC) obtained from FRDA patients. Compound **106** (also called RGFA8) that had previously shown efficacy in this system was used as benchmark positive control. Compound **136** showed a limited effect in this test (Figure 2A), even at the highest tested concentration of 10 µM, when compared to 10 µM compound **106**. Frataxin mRNA was not affected by **136** treatment in PBMC samples P1, P2 and P6, and was mildly upregulated in samples P3-P5 and P7, whereas compound **106** increased frataxin mRNA levels by 3.6 fold on average (*p*<0.001 in all tested patients with compound **106**). Conversely, compound **109** was highly active in the whole tested concentration range from 1 to 10 µM (Figure 2B) (*p*<0.001 in all tested patients with compound **109** at 10 µM), showing an almost linear dose-response relation with the induced frataxin mRNA increase, except in the case of patient P12. This individual is a late-onset, mildly affected FRDA patient, who carries relatively short GAA repeat expansions and has higher baseline frataxin levels than the other patients, suggesting that HDAC inhibition may be more effective when long GAA repeat strongly downregulate *FXN* expression. Healthy volunteers PBMCs were not overall significantly affected by either treatment, though non-significant fluctuations and a trend toward downregulation by compound **136** were observed (Figure S3). These data are in agreement with our previous observation that no frataxin upregulation is induced by compound **106** in the brain and heart of wild-type mice [8]. They indicate that the upregulation of frataxin levels induced by these compounds in patients' cells is due to an effect on the epigenetic changes caused by the expanded GAA repeat.

To follow the duration of effect of **109** in PBMC, cells were treated with 10 µM **109** or, as a control, with its dimethylsulfoxide (DMSO)-containing buffer for 24, 48 or 72 hours. Frataxin mRNA increased to 2.4-fold after 24 hours, 2.7-fold after 48 hours and 1.8-fold after 72 hours, as revealed by quantitative RT-PCR (Figure 2D). In a second set of experiments, cells from patient P13 were treated for 48 hours with 10 µM **109** or DMSO. A fraction of cells was collected and another fraction was washed and sub-cultured in regular media without the HDAC inhibitor for further 6, 12, or 24 hours (Figure 2F). Frataxin mRNA levels were still stable 6 hours after removing the drug, they showed an initial decrease at 12 hours and returned to the pre-treatment baseline 24 hours after drug removal. The corresponding increase in frataxin protein, as determined by western blot, occurred more slowly, reaching ∼2 to ∼3.6 fold over baseline (in two separate experiments) at 48 to 72 hours of continuous **109** incubation (Figure 2E and 2G). When the compound was removed after 48 hours of incubation, frataxin protein levels rapidly increased up to ∼6 fold over baseline at 6 hours after the drug washout, and this marked increase was still present at 24 hours (Figure 2G). The increase in *FXN* gene expression was paralleled by increased histone acetylation, both when determined globally by estimating the ratio between acetylated and total histone H3, and locally near the *FXN* GAA repeat by quantitating acetylation of histone H4 at lysine 5 (H4K5) by chromatin immunoprecipitation (ChIP) (Figure S3).

### Effects of Compounds 109 and 136 in KIKI Mice

**Figure 3:**
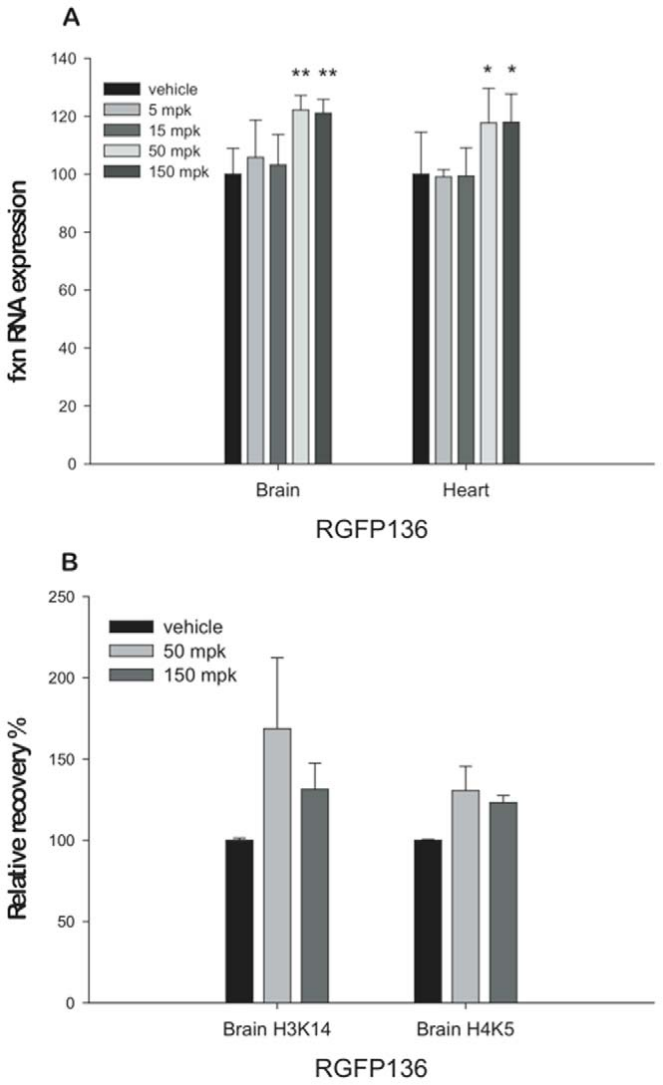
Frataxin expression changes as function of drug 136 dosage. **A.** *Fxn* mRNA measurement by quantitative real-time RT-PCR in brain and heart of KIKI mice dosed subcutaneously with **136** at 5 (n = 7), 15 (n = 7), 50 (n = 6) or 150 (n = 6) mg/kg or vehicle (n = 13) as negative control. *Fxn* levels were relative to RER1 and beta-2 microglobulin (β2M). The *Fxn* level in vehicle injected KIKI of each tissue was set to 100. All quantifications were done in triplicate and bars indicate s.d. (** *p*<0.001; * *p*<0.05). **B.** Levels of H4K5 and H3K14 acetylation in KIKI mice brain treated with one subcutaneous injection of 50 or 150 mg/kg of **136** compared to vehicle-treated KIKI littermates (n = 4). We performed ChIP experiments with antibodies for murine histones H3 and H4 carrying each modification. Primer pairs corresponded to the first intron of the mouse frataxin gene just upstream of the point of insertion of the GAA repeat in KIKI mice. Relative recovery, determined by qPCR, is expressed in relation to GAPDH and the recovery in samples from vehicle treated KIKI animals is set to 100 for each antibody. Each immunoprecipitation was quantified in triplicate, Error bars are s.e.m.

We previously showed that homozygous knock-in mice carrying a (GAA)_230_ repeat in the first intron of the endogenous frataxin gene (*fxn*), called KIKI mice [16], recapitulate the genetic and epigenetic changes observed in FRDA [9]. We injected a group of KIKI mice (n = 5 per group) with a single subcutaneous dose of 150 mg/kg of compound **136** or with an equivalent volume of vehicle. We sacrificed the mice 24 hours after the injection and found an increase in frataxin mRNA in the brain to wild-type levels. To determine the lowest dose of **136** that could achieve a similar frataxin mRNA increase, we injected KIKI mice with a single subcutaneous dose of 5, 15, 50 or 150 mg/kg of compound **136** (n = 6 or 7 per group) or with vehicle (n = 13). *Fxn* mRNA significantly increased in the brain (p<0.001) and in the heart (p = 0.009 and 0.008 respectively) only in mice that received 50 or 150 mg/kg (Figure 3A). In the brains of mice from these groups, we observed by chromatin immunoprecipitation (ChIP) an increase in histone acetylation at positions H3K14 and H4K5 in the region of the *fxn* gene immediately upstream of the inserted GAA repeat (Fig. 3B).

**Figure 4:**
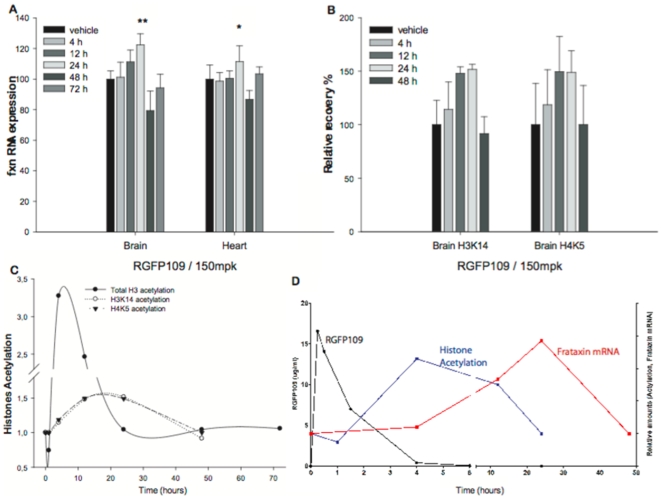
Time course for drug exposure, frataxin mRNA expression, and histone acetylation changes in the brain and heart of KIKI mice. **A.** *Fxn* mRNA measurement by quantitative real-time RT-PCR in brain and heart of KIKI mice dosed subcutaneously with **109** at 150 mg/kg (n = 26) or vehicle (n = 13). Treated mice were sacrificed at one of the mentioned time points: 4, 12, 24, 48 or 72 h. Vehicle treated mice were sacrificed 24 hours after injection. *Fxn* mRNA levels were relative to RER1 and beta-2 microglobulin (b2M). The *Fxn* level in vehicle injected KIKI of each tissue was set to 100. All quantifications were done in triplicate and bars indicate s.d. (** *p*<0.001; * *p*<0.05). **B.** Levels of H4K5 and H3K14 acetylation in KIKI mice brain treated with one subcutaneous injection of 150 mg/kg of **109** compared to vehicle-treated KIKI littermates, after 4, 12, 24 or 48 hours (n = 4 for each shown time point). We performed ChIP experiments with antibodies for murine histones H3 and H4 carrying each modification. Primer pairs corresponded to the first intron of the mouse frataxin gene just upstream of the point of insertion of the GAA repeat in KIKI mice. Relative recovery, determined as previously (Fig. 3B) is set to 100 for each residue. Each immunoprecipitation was quantified in triplicate. Error bars are s.e.m. **C.** Brains from wild-type mice for total histone H3 acetylation and from KIKI mice for specific residues acetylation, injected with either vehicle or with 150 mg/kg of **109**, were harvested after a period of 1, 4, 12, 24, 48 or 72 hours (n = 5 per condition) and subjected to western blot with total or acetylated histones H3 antibodies for WT samples or to chromatin immunoprecipitation for KIKI samples. Ratio of acetylated H3 over total H3 is plotted, showing a peak of acetylation at 4 hours. Recovery of specific residues is plotted, showing a plateau between 12 and 24 hours. **D.** Composite graphic of **109** measures in mouse brain: in black, drug **109** exposure measured in µg/ml; in blue, total histone acetylation from western blot quantification (panel C); in red, *fxn* RNA as determined by qPCR in panel A.

Compound **109** was also able to correct frataxin deficiency in the brain and heart of KIKI mice 24 hours after a single injection of a 150 mg/kg dose, but not when lower doses were used (data not shown). When followed in time, the frataxin mRNA increase induced by compound **109** in the KIKI mouse could be first detected at 12 hours (n = 4) and reached a maximum at 24 hours (n = 9) in both brain (p<0.001) and heart (*p* = 0.02) (Figure 4A). Frataxin mRNA levels then showed a decrease at 48 hours and returned to normal levels by 72 hours. The frataxin protein similarly increased at 24 hours and returned to baseline at 72 hours (data not shown). Histone acetylation of residues H3K14 and H4K5 in the region upstream of the GAA repeat, as revealed by ChIP, also increased between 12 and 24 hours after the HDACI injection (Figure 4B). In order to compare this effect on histone acetylation near the GAA repeat with the overall effect of the compound on histone acetylation, we injected a group of wild-type mice (n = 5 per time point) with either vehicle or **109** at 150 mg/kg and estimated total and acetylated histone H3 levels in brain by western blot (Figure 4C). Acetylation increased to a maximum level at 4 hours after injection and totally disappeared at 24 hours. Finally, we determined the levels of compound **109** in the mouse brain and plasma after a single 150 mg/Kg injection and found out that it is totally eliminated before 4 hours (Figure 4D). No acute toxicity signs such as changes in respiration, activity, temperature or lack of sedation were noticed in any of the above experiments, as observed in the open cage behavior.

**Figure 5:**
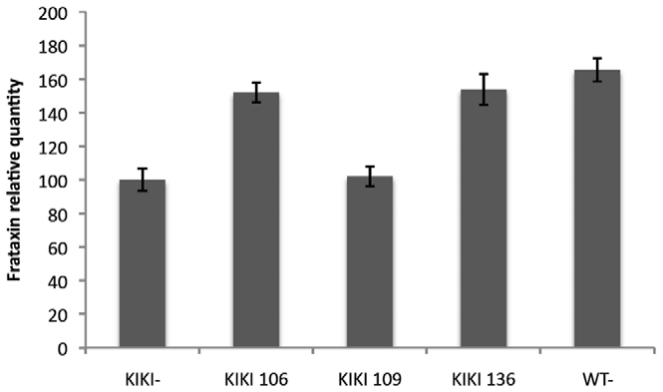
Effect of compounds 106, 109 and 136 in unstimulated KIKI splenocytes. Splenocytes were collected from pooled KIKI spleens (n = 3) or WT spleens. Spleen mononuclear cells were cultured in triplicate for 48 hours with one of HDACi **106**, **109** and **136** at 10 µM or with DMSO for KIKI samples. WT samples were cultured in triplicate for 48 hours with. Quantitative real-time RT-PCR was used to determine relative *Fxn* levels for each condition using *RER1* and β2M as control housekeeping genes. DMSO KIKI condition was set to value 1. Error is represented as the standard deviation of the mean from 3 determinations. *p*<0.001 for **106** and **136** KIKI responses.

Because of the discrepancy between patients' PBMCs and KIKI mouse tissues in the relative response to compounds **109** and **136**, we compared the effect of HDACi **106**, **109** and **136** in splenocytes collected from pooled KIKI or WT mice spleens. Splenocytes are close to PBMCs in terms of cell type composition, both being mostly lymphocytes, allowing to test whether the different response in human vs. mouse was due to a species difference or to a different relative sensitivity to the HDACIs of the tested cell types, mostly lymphocytes in humans vs. central nervous system and heart cells in the mouse. In mouse splenocytes, frataxin mRNA upregulation was significant (p<0.001) only with compounds **106** and **136** (Figure 5), supporting a species difference.

## Discussion

We generated new pimelic diphenylamide HDACIs to maximize efficacy in upregulating frataxin and to optimize pharmacological properties in view of a future translation to the clinic. The new compounds show the same type of inhibition kinetics and HDAC3 specificity as the previously reported molecules. We selected two of these compounds, **109** and **136**, that differ for overall potency and degree of specificity for HDAC3, both being higher for compound **109** (Figure 1), and tested them in FRDA patients' PBMC and in the KIKI mouse model. We then determined in both systems the duration of effect of these drugs on frataxin mRNA and protein, and on total and local (upstream of the GAA repeat) histone acetylation.

In FRDA patients' PBMC, compound **109** was much more potent than compound **136** in upregulating frataxin, supporting the concept that HDAC3 selectivity correlates with this property. A good dose-response correlation could be detected for compound **109**, with a 4- to 9-fold upregulation of frataxin mRNA at the highest tested concentration of 10 µM. Rather surprisingly, however, compound **136** turned out to be effective at a threefold lower dose and more consistently in the KIKI mouse model, both in the FRDA target tissues after sub-cutaneous treatment and in *in vitro* treated splenocytes. At this time we have no definite explanation for such a discrepancy. The similar response of mouse splenocytes (closely related to the PBMCs tested in human patients) and of mouse nervous and cardiac tissue supports the hypothesis of a species difference rather than a cell type difference. It is possible that the mouse and human enzymes have differences in the response to the inhibitor, the inhibition kinetics having been determined with the human enzyme. It is also possible that the compounds differ in pharmacokinetic or metabolic properties in the mouse. Such species differences have to be taken into account in the preclinical development of these drugs.

An important conclusion of this study comes from the analysis of the time course of the effects of these compounds in patients' PBMC and in mice, that long exceeds the time of direct exposure to the drugs. Frataxin mRNA remained elevated in PBMC for at least 12 hours after drug removal and in mice for at least 24 hours after a single subcutaneous injection. In both systems, the time course of frataxin mRNA upregulation paralleled the increase in total and local histone acetylation. These data confirm that HDAC inhibition is a more persistent phenomenon after transient exposure to this category of compounds than to other HDACIs like SAHA [15]. Most importantly, the increase in frataxin protein was the most sustained effect, lasting more than 48 hours after drug dosing in the mouse. It is of note that frataxin protein levels sharply increased in PBMC immediately after drug washout, reaching much higher levels compared to those obtained with continued exposure to the compounds. This finding may be due to inhibition of translation or of some post-transcription RNA processing step by the HDACI [17]. Taken together, these data suggest that intermittent administration of the drug, i.e. less than once daily and possibly as spaced as once or twice a week, may be the dosing regime of choice for the future clinical testing of these drugs. Such regimen would minimize toxic side effects by reducing drug exposure, at the same time allowing sustained upregulation of frataxin protein.

Finally, it is important that this class of HDACIs has not shown any apparent toxicity in this series of experiments, neither in cell culture, nor in the animal studies. These studies were not designed to provide a complete assessment of acute toxicity, however, and complete preclinical testing will be necessary before any human test. Together with the previous studies [8], [9], the present results support the pre-clinical development of pimelic diphenylamide HDACIs as potential therapeutics for FRDA.

## Methods

### Ethics Statement

Friedreich's ataxia patients donated blood (Human Subjects Protocol approved by the Erasme Hospital ethics committee) with appropriate written informed consent. All animal procedures respected regulations and guidelines of the Belgian state and European Union and were approved by the local ethical committee (CEBEA).

### Animal Procedures

GAA knock-in mice were generated and genotyped as described[16]. Age and gender matched WT littermates were used as controls. Mice were treated by a single subcutaneous injection with 150 mg/kg (or other dose when mentioned) of HDACi **109** or HDACi **136** or its equivalent of vehicle. Brain and heart were recovered 24 hours after last injection, or at indicated time.

### Primary Lymphocytes Culture

About 40 ml of blood was collected in heparinized Vacutainer tunes (BD Biosciences) and lymphocytes were isolated by density centrifugation using Ficoll – Paque PLUS (GE Healthcare), according to the manufacturer. Lymphoctyes were maintained in RPMI 1640 medium with 2 mM l-glutamine, penicillin / streptomycin and 15% FBS at 37°C in 5% CO_2_. Cells viability and morphology were monitored by trypan blue exclusion on phase-contrast microscopy. We dissolved compounds **106**, **136** and **109** in DMSO and added the solution into the culture medium at the concentrations and for the duration indicated in Figure 2. Controls were treated with the same concentration of DMSO-lacking compounds.

### Mouse Splenocytes Culture

Spleens collected from KIKI or WT mice were mashed between 2 glass slides, ground with a 1-mL syringe pestle, then suspended in RPMI 1640 medium supplemented with 2 mM l-glutamine, 15% fetal calf serum and penicillin / streptomycin before filtering through a 75-µm nylon mesh. After washes, red blood cells were removed with lysis buffer (155 mM NH_4_Cl (pH 7.4), 10 mM NaHCO_3_, 0.1 mM EDTA). Spleen mononuclear cells were collected, washed, and resuspended before subculturing in triplicate for 48 hours in culture medium containing DMSO (negative control (-)) or one of HDACi **106**, **109** and **136** at 10 µM.

### Quantitative Real Time PCR

Total RNA was extracted from PBMC and splenocytes by RNeasy Mini Kit (Qiagen). Total RNA from mice brain was extracted by RNeasy Lipid Tissue Mini Kit (Qiagen) as recommended by the manufacturer. RNA from mice heart was extracted by RNeasy Fibrous Tissue Mini Kit (Qiagen). All RNA samples were treated with RNase-Free DNase Set (Qiagen) and quantified afterwards by measuring the optical density (NanoDrop ND-1000 Spectrophotometer, NanoDrop Technologies). Quality and purity of some samples were analyzed by gel electrophoresis on RNA assay chips (Experion System and StdSens analysis kit, BioRad Laboratories). We performed one-step qRT-PCR using MultiScribe Reverse Transcriptase with Power SYBR Green (both from Applied Biosystems). Primers used for *FXN* were 5′-CAGAGGAAACGCTGGACTCT-3′ and 5′-AGCCAGATTTGCCTTGTTTGG-3′, and primers for *Fxn* were 5′- CCTGGCCGAGTTCTTTGAAG-3′ and 5′- GCCAGATTTGCTTGTTTGG-3′. Cells RNA was standardized by quantification of GAPDH. Mice brain and heart RNA was quantified relative to *RER1* and beta-2 microglobulin (β2M) using qBase 1.3.4 (Jan Hellemans & Jo Vandesompele). These control genes were chosen because they did not show genotype (KIKI vs. wild-type) or treatment-related changes. Data are normalized to the *fxn* mRNA level in vehicle treated KIKI ( = 100%) or to the *FXN* (or *Fxn*) RNA level in DMSO treated cells ( = 1).

### Western Blot Analysis

Tissues were homogenized in T-PER tissue protein extraction reagent (Pierce) for total proteins extraction. Histones were purified by acid extraction as described in the protocols from Upstate. Primary antibodies were diluted in Odyssey blocking buffer (Li-Cor) for frataxin (Chemicon) and actin (Sigma) or in PBS for total and acetylated histones antibodies (Upstate) and for human frataxin (Mitosciences). Infrared dye conjugated secondary antibodies (anti-rabbit IRdye800Cw and anti-mouse IRdye680 from Li-Cor) were used to detect and quantify the signal of mouse frataxin / actin using a Li-Cor Odyssey imaging system. Horseradish Peroxidase (HRP) - conjugated secondary antibodies (Santa Cruz Biotechnology) were used to detect the signal of total and acetylated histones and for the human frataxin / Gapdh signal by chemiluminescence.

### Chromatin Immunoprecipitation

ChIP was performed as described previously[20]. We adapted the technique for use on fresh brain tissue and were able to test 3 different residues per one hemisphere. For each immunoprecipitation, lysate was incubated with one of the following antibodies (Anti-acetyl-Histone H3 (Lys14): Upstate Biotechnology 07-353; Anti-acetyl-Histone H4 (Lys5): Upstate 07-327; Normal Rabbit IgG: Upstate 12-370). Immunoprecipitated samples were quantified by real-time PCR following the standard curve method. Primers used for the region upstream intronic GAA repeats of *Fxn* were 5′- ACGACAAAGTCTCCCACAGG-3′ and 5′- GTCCAACAAGGCTTGATTCC-3′. GAPDH primers were: 5′-TGGGTGGAGTGTCCTTTATCC-3′ and 5′-TATGCCCGAGGACAATAAGG-3′.

### Statistical Analyses

Data are presented as mean ± s.d. or s.e.m., or as percentages. The significance of the difference between groups was evaluated with the Student's *t*-test or one-way ANOVA. *p*<0.05 was considered significant. (*p<0.05 unless otherwise noted).

## Supporting Information

Figure S1 Compound 136 IC50 determination for HDAC1 and HDAC3/NcoR2 (left panel, top/bottom). Compound 136 shows a time-dependent inhibition of HDAC3/NcoR2. IC50s against HDAC3/NcoR2 decrease from 16.8 µM to 560 nM over a period of 3 hours. There is no time-dependent inhibition of HDAC1 with compound 136. Compound 136 is a fast on/off inhibitor of HDAC1 with Ki at 630 nM (right panel, top). Compound 136, however, is a slow-tight binding inhibitor of HDAC3/NcoR2 with Ki at 196 nM (right panel, bottom).
(2.63 MB TIF)

Figure S2 Inhibitor 109 IC50s determination for HDAC1 and HDAC3/NcoR2 (left panel, top/bottom). Inhibitor 109 shows a time-dependent inhibition for both HDAC1 and HDAC3/NcoR2. Its IC50s against HDAC1 decrease from 230 nM to 60 nM within an hour. Inhibitor 109 IC50s against HDAC3/NcoR2 decrease from 1 µM to 50 nM over a period of 3 hours. 109 are slow-tight binding inhibitor of both HDAC1 and HDAC3/NcoR2, but the on rate of 109 is faster for HDAC1 than for HDAC3/NcoR2. Compound 109 has a Ki of 32 nM (right panel, top) for HDAC1 and a Ki of 5 nM for HDAC3/NcoR2 (right panel, bottom).
(2.21 MB TIF)

Figure S3 Upper panel: Effect of compounds 106, 109 and 136 on PBMCs from subjects with normal GAA repeats (healthy volunteers). FXN mRNA levels were measured after a 48-hour incubation with either 106, 109 and 136 at 10 µM or DMSO (at 0.1%). Quantitative real-time RT-PCR was used to determine relative FXN mRNA levels for each individual condition using GAPDH as control housekeeping gene. DMSO condition was set to value 1 for each individual. Error is represented as the standard deviation of the mean from three dterminations. PBMCs were obtained from donors' blood as described [8]. Middle and lower panels: Changes in local and global acetylation upon removal of HDACi 109. PBMCs from patient P13 were treated with either DMSO or 109 for 48 hours and part of these two samples were collected (samples “DMSO” and “0h”), and the rest washed to remove the inhibitor. Samples were then collected at 6, 12, 24 and 48 hours after washing. At each time point, we measured FXN mRNA levels (upper left panel), occupancy of H4AcK5 on the frataxin gene, upstream of the GAA repeats (upper right panel) and global H3 acetylation (lower left panel, quantified in the lower left panel).
(2.55 MB TIF)

Methods S1 Supplementary Methods.
(0.04 MB DOC)
