# Supplementary figures and images for: Two New Pimelic Diphenylamide HDAC Inhibitors Induce Sustained Frataxin Upregulation in Cells from Friedreich's Ataxia Patients and in a Mouse Model
Source: PLoS One. 2010 Jan 21;5(1):e8825. doi: 10.1371/journal.pone.0008825 (PMC2809102; doi:10.1371/journal.pone.0008825)

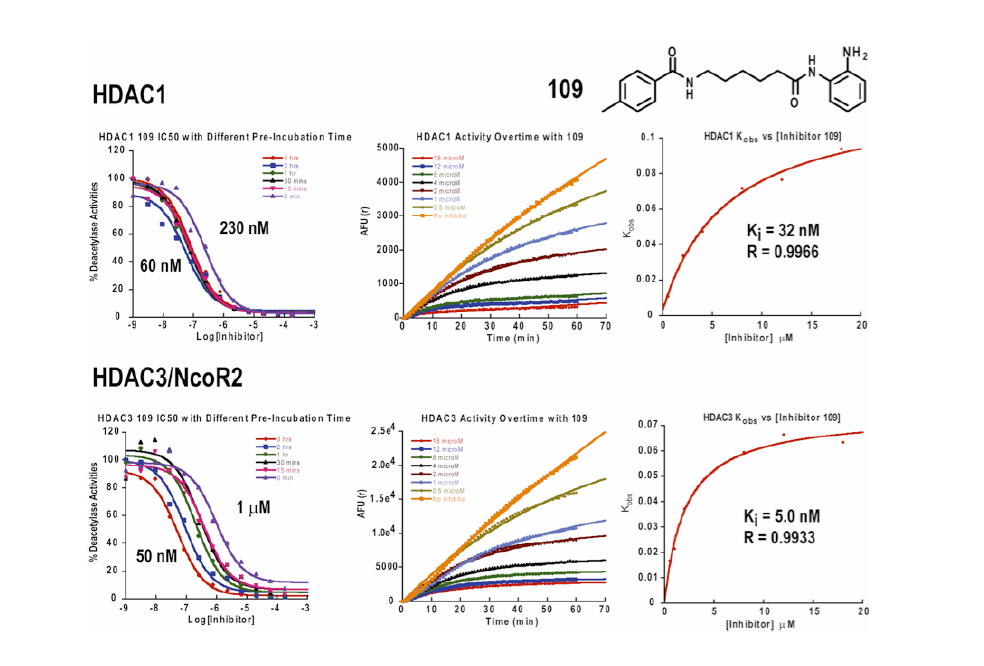

Supplement: Figure S1 — Compound 136 IC50 determination for HDAC1 and HDAC3/NcoR2 (left panel, top/bottom). Compound 136 shows a time-dependent inhibition of HDAC3/NcoR2. IC50s against HDAC3/NcoR2 decrease from 16.8 µM to 560 nM over a period of 3 hours. There is no time-dependent inhibition of HDAC1 with compound 136. Compound 136 is a fast on/off inhibitor of HDAC1 with Ki at 630 nM (right panel, top). Compound 136, however, is a slow-tight binding inhibitor of HDAC3/NcoR2 with Ki at 196 nM (right panel, bottom). (2.63 MB TIF) [file pone.0008825.s001.tif]

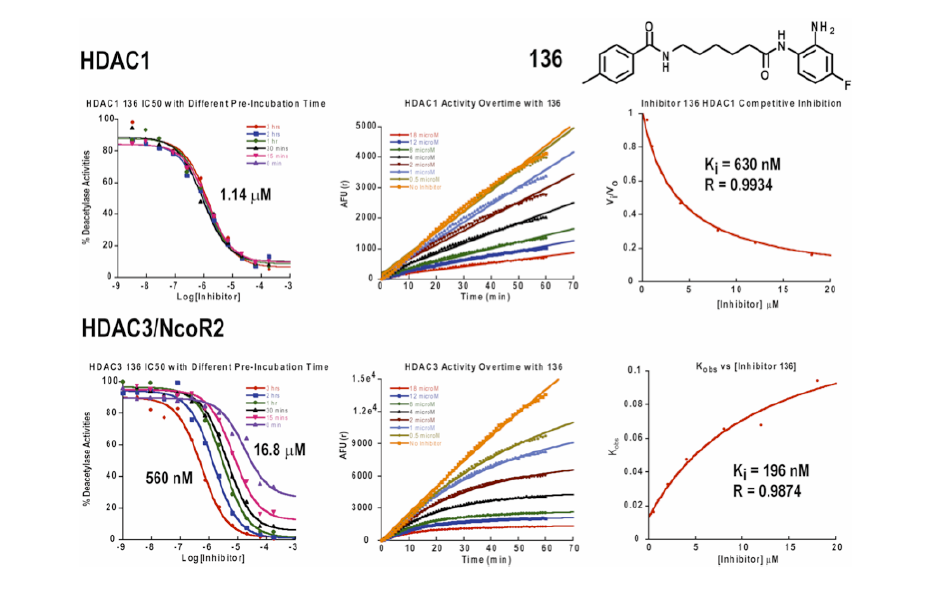

Supplement: Figure S2 — Inhibitor 109 IC50s determination for HDAC1 and HDAC3/NcoR2 (left panel, top/bottom). Inhibitor 109 shows a time-dependent inhibition for both HDAC1 and HDAC3/NcoR2. Its IC50s against HDAC1 decrease from 230 nM to 60 nM within an hour. Inhibitor 109 IC50s against HDAC3/NcoR2 decrease from 1 µM to 50 nM over a period of 3 hours. 109 are slow-tight binding inhibitor of both HDAC1 and HDAC3/NcoR2, but the on rate of 109 is faster for HDAC1 than for HDAC3/NcoR2. Compound 109 has a Ki of 32 nM (right panel, top) for HDAC1 and a Ki of 5 nM for HDAC3/NcoR2 (right panel, bottom). (2.21 MB TIF) [file pone.0008825.s002.tif]

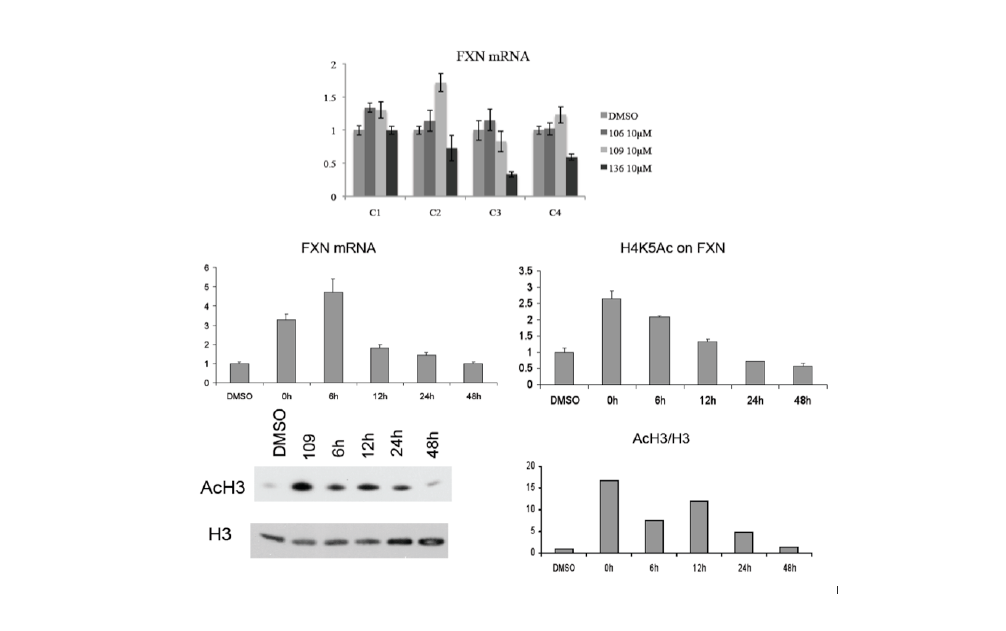

Supplement: Figure S3 — Upper panel: Effect of compounds 106, 109 and 136 on PBMCs from subjects with normal GAA repeats (healthy volunteers). FXN mRNA levels were measured after a 48-hour incubation with either 106, 109 and 136 at 10 µM or DMSO (at 0.1%). Quantitative real-time RT-PCR was used to determine relative FXN mRNA levels for each individual condition using GAPDH as control housekeeping gene. DMSO condition was set to value 1 for each individual. Error is represented as the standard deviation of the mean from three dterminations. PBMCs were obtained from donors' blood as described [8]. Middle and lower panels: Changes in local and global acetylation upon removal of HDACi 109. PBMCs from patient P13 were treated with either DMSO or 109 for 48 hours and part of these two samples were collected (samples “DMSO” and “0h”), and the rest washed to remove the inhibitor. Samples were then collected at 6, 12, 24 and 48 hours after washing. At each time point, we measured FXN mRNA levels (upper left panel), occupancy of H4AcK5 on the frataxin gene, upstream of the GAA repeats (upper right panel) and global H3 acetylation (lower left panel, quantified in the lower left panel). (2.55 MB TIF) [file pone.0008825.s003.tif]
