# Supplementary material for: Two New Pimelic Diphenylamide HDAC Inhibitors Induce Sustained Frataxin Upregulation in Cells from Friedreich's Ataxia Patients and in a Mouse Model
Source: PLoS One. 2010 Jan 21;5(1):e8825. doi: 10.1371/journal.pone.0008825 (PMC2809102; doi:10.1371/journal.pone.0008825)
Supplement: Methods S1 — Supplementary Methods. (0.04 MB DOC) [file pone.0008825.s004.doc]

Supplementary materials and methods

**Time dependent IC50 value with pre-incubation** – Deacetylation assays were based on the homogenous fluorescence release assay as previously described [17]. In brief, purified recombinant enzymes were incubated with serial-diluted inhibitors at the concentrations indicated in the figures, with pre-incubation times ranging from 0 to 3 hours, in the standard HDAC buffer. Acetyl-Lys(Ac)-AMC substrate (at 10 μM, corresponding to the Km for both HDAC1 and HDAC3) was added after the pre-incubation period. The reaction was allowed to run for 1 hour. The trypsin peptidase developer, at final concentration of 5mg/ml, was added after 1 hour, and the fluorescence emission was then measured using a Tecan M200 96-well plate reader (San Jose, CA).

**Enzyme kinetic determination using the progression method** – Both fast on/off and slow tight-binding kinetics of all inhibitors with HDAC1 and HDAC3/NcoR2 were evaluated by the progression method approach described previously [17,21]. The reaction mixture contained 100 ng of enzyme in the standard HDAC Tris buffer (pH 8.0), 50 μM acetylated lysine substrate, and various concentrations of inhibitor **136** (indicated), and 2 milliunits of Lys-C peptidase (EMD, San Diego, CA) were prepared and monitored every minute for 60 minutes. The curves were then fitted to determine vi/vo (fast on/off inhibition) or Kobs (slow tight-binding inhibition) through the appropriate equations. The Ki was then determined through either vi/vo or Kobs verse the inhibitor concentration as described previously [17].
